# Differential Binary Encoding Method for Calibrating Image Sensors Based on IOFBs

**DOI:** 10.3390/s120404133

**Published:** 2012-03-27

**Authors:** Pedro R. Fernández, José Luis Lázaro-Galilea, Alfredo Gardel, Felipe Espinosa, Ignacio Bravo, Ángel Cano

**Affiliations:** 1 Department of Electronics, University of Alcalá, Alcalá de Henares, 28871 Madrid, Spain; E-Mails: pedro.fernandez@depeca.uah.es (P.R.F.); alfredo@depeca.uah.es (A.G.); felipe@depeca.uah.es (F.E.); ibravo@depeca.uah.es (I.B.); 2 Department of Telecommunications and Electronics, University of Oriente, Santiago de Cuba, 90400, Cuba; E-Mail: acano@fie.uo.edu.cu

**Keywords:** image sensor, image transmission, sensor calibration, optical fiber sensors

## Abstract

Image transmission using incoherent optical fiber bundles (IOFBs) requires prior calibration to obtain the spatial in-out fiber correspondence necessary to reconstruct the image captured by the pseudo-sensor. This information is recorded in a Look-Up Table called the *Reconstruction Table* (RT), used later for reordering the fiber positions and reconstructing the original image. This paper presents a very fast method based on image-scanning using spaces encoded by a weighted binary code to obtain the in-out correspondence. The results demonstrate that this technique yields a remarkable reduction in processing time and the image reconstruction quality is very good compared to previous techniques based on spot or line scanning, for example.

## 1. Introduction

Visual inspection systems based on electronic cameras are widely used these days for quality control in various industrial processes and for surveillance systems, positioning and identification of mobile objects and robotics, *etc.* The majority of systems based on artificial vision have been designed for a specific application and thus lack the flexibility necessary for use in other environments where the use of electrical signals or electronic devices may not be possible or suitable. Examples of these include environments which are difficult to access because they are winding and/or narrow, medical applications linked to endoscopy, and the inspection of hostile environments exposed to high temperatures, the risk of explosion, corrosion, the presence of radiation, *etc*. To transmit images under these conditions, coherent optical fiber bundles can be used, where the fibers maintain the same spatial relationship (or position) with respect to one another. In this way, it is possible to achieve more effective physical access to the target medium, and high galvanic isolation is assured.

In a fiber bundle, any image projected onto the input plane of the bundle is broken down into different points related to the image plane, appearing at the output as a set of luminous points transmitted by each fiber. Most present day applications using coherent fiber bundles to transport images only permit transmission over short distances and at a relatively high cost *per* meter length, which can limit their range of uses in remote environments. In contrast, incoherent optical fiber bundles (IOFBs) are generally used as light guides although under certain conditions they can also be used to transmit images, and constitute a cheaper medium that can attain a greater working distance. Since, from a production point of view, fiber distribution in these devices is less exacting, their cost is considerably lower. Furthermore, in contrast to coherent bundles, the fibers are not subjected to a fusion process to reduce the interstitial spaces between them. Thus, it is possible to obtain greater flexibility and less inter-fiber crosstalk, which can be a possible cause of contrast loss in the received image [1].

**Figure 1.**
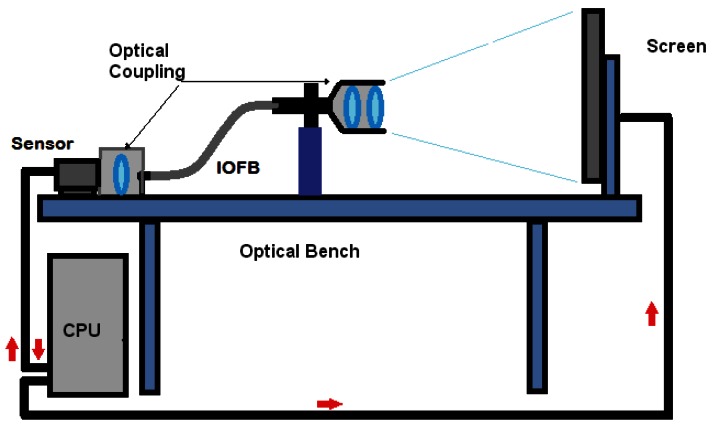
Diagram of an image calibration/transmission system based on IOFBs.

A system with these characteristics requires a sensor or camera connected to a processing unit that “decodes” the information received at the bundle output, since this is naturally “encoded” due to the random distribution of the fibers. This implies that in order to transmit and reconstruct images with IOFBs, it is necessary to calibrate the system before transmission in order to estimate the transfer function necessary between input and output to recover the information captured [2–5]. An image calibration/transmission system based on IOFBs is generally composed of the elements shown in Figure 1 [4–6]. Both the sensor and the calibration screen are controlled from a central processing unit (CPU), which is also involved during the process of capturing and reconstructing the final image.

In brief, the calibration procedure consists of scanning the bundle input end with appropriate pattern images projected from a screen. In this way, the input-output transfer function of the system is determined, verifying the effect produced by the set of pattern images on each fiber at the output end. The pattern images used strongly influence the speed of the calibration process and the quality of the results obtained, and can be formed by square pixel regions [7], luminous lines [4,6] or encoded images of the bundle [8] in both vertical and horizontal dimensions.

In [8], a calibration method is presented in which a series of encoded pattern images was used. The authors stressed the need to previously locate the fibers in order to determine beforehand where the useful information would be extracted from during the calibration procedure. This problem was solved using the simple procedure described in [3,4], extending its application to the process of reconstructing and correcting the transmitted images, which is extremely useful regardless of the scanning method employed.

### Calibration of IOFBs by Means of the Space Encoding Technique

For calibration, in [8] the input end of the bundle was scanned with pattern images composed of areas of high contrast (black and white) consisting of vertical or horizontal lines, in such a way that with each scan, approximately half of the fiber bundle was illuminated. This technique, known as *space encoding*, is frequently employed to reconstruct 3D environments [9,10]. The pattern images are generated using a binary code, and this is an efficient form of scanning the input end of the bundle. When the behavior of each fiber at the bundle output in response to each of the pattern images is known, the corresponding positions at the input end can be calculated and this information is stored in a *reconstruction table* (RT). The input/output relationship is achieved with a notable reduction in processing time compared to square region scanning techniques [7] or luminous line techniques [6], and the number of images required is also notably reduced.

**Figure 2.**
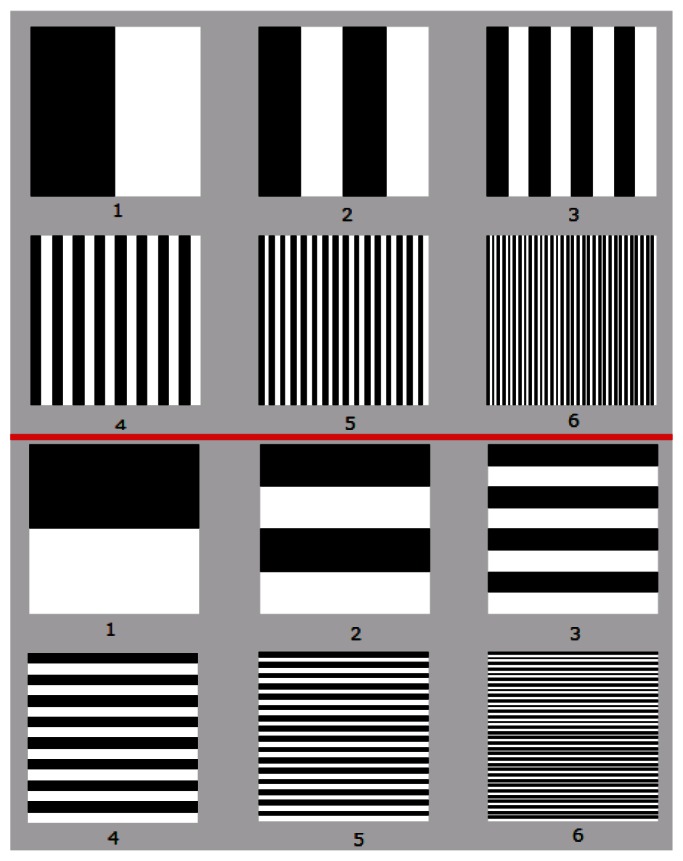
An example showing a set of pattern images according to the space encoding scan described in [8].

Figure 2 gives an example of pattern images as proposed in [8], but only shows six different images for each dimension (x and y). The pattern images consist of multiple black and white lines, the structure of which (width and position) is determined by a weighted binary code. Each space dimension of a discrete scan is subdivided by “*n*” areas of excitation. Therefore, given that the base which generates the pattern images for the horizontal and vertical dimensions is binary, a total of 2log_2_(n) images are required. For example, in [8], a bundle of approximately 256 × 256 fibers was used, requiring a total of at least 16 encoded images; eight to scan the horizontal dimension and another eight to scan the vertical one.

The degree of focus, aberrations and the resolution of the optic used in the input subsystem can all produce some *blurring* on the images which impacts on the input and therefore can decisively affect the quality of calibration. If these questions are not taken into account, the incident energy may be scattered. For example, when the focus of the input optic is incorrect, the incident energy in those regions where abrupt changes of intensity should occur (dark to light or *vice versa*) will be scattered toward adjoining areas, rendering estimation of the state of the fibers in response to a given pattern image complicated. Furthermore, as the image appears disordered at the output, the focusing process implies another additional difficulty since the real structure of the transmitted image is lost; consequently, some traditional focus methods are not applicable. In [2], a focus methodology is proposed which uses simple metrics and ensures a notable improvement in calibration results.

In the present article, we describe a novel calibration procedure for remote visual inspection systems based on IOFBs which employs a scanning method with differentially encoded images (differential space encoding—DSE) and yields short processing times and guarantees fewer of the calibration errors fundamentally generated by the problems mentioned above.

## 2. Experimental Section

### 2.1. Model of the Scanning Space and the RT Structure

**Figure 3.**
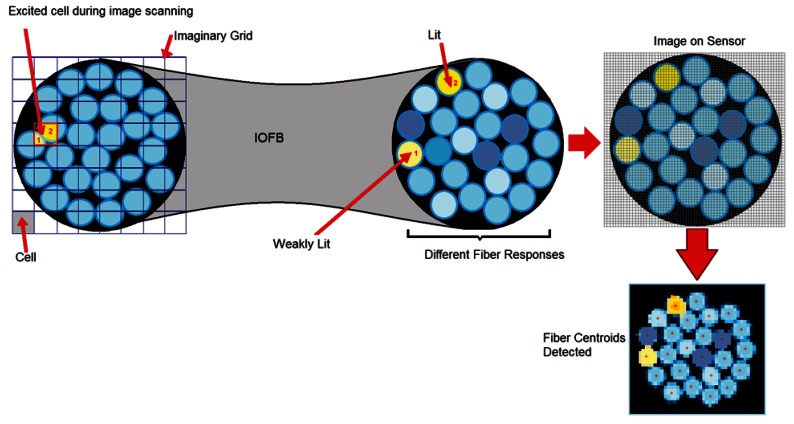
Relationship between the imaginary grid cells and their effect on the sensor.

Before presenting the proposed method, it is necessary to define a model for the calibration space required. Given that fiber distribution at the input is irregular, the input is subdivided into different square regions which we will call cells. This set of cells comprises a kind of imaginary grid (see Figure 3) that defines a 2D space of discrete scanning. It is on this plane that the set of appropriately selected pattern images will impact, being projected from the calibration screen. Any scanning procedure should be capable of generating a sequential set of unique and predefined images that will pass through and excite all the cells in the imaginary grid. Each side (*l*) of a cell has a length almost equivalent to the average diameter *d_fib_* of the fibers, such that 3/4 *d_fib_* ≤ *l* ≤ *d_fib_*. This does not it imply that an exact correspondence can be established between each fiber and each cell since the area of influence of a cell can cover more than one fiber. Nevertheless, a cell can be associated with the fiber that receives its greatest area of influence (see Figure 3).

Although fundamentally spatial, this calibration will not only have to take geometric parameters into account, but should also include an implicit calibration of the fiber responses since the information that is extracted is always affected by the transfer functions of the fibers themselves (attenuation) or by the input optic. Therefore, these responses must be compensated for in such a way that all the pixels in the image to be formed possess equal gray levels. An exhaustive analysis of this problem is given in [6] and has been applied in the present study.

According to the restrictions imposed by the model, a RT is proposed that has a maximum number of entries defined by the number of “locatable” fibers in the bundle image captured. In each RT row or entry, the centroid of a located fiber is associated with the position of a cell at the input end and also with an equalization factor associated with that fiber. The centroid of each fiber *i* refers to the 2D coordinate system of the camera [r(*i*), c(*i*)] and represents the discrete position, in the image to be reconstructed, to which the information extracted from the central region of a determined fiber should be transferred. The associated cell will be the position with the maximum probability of guaranteeing that the fiber assigned will attain greatest *emittance* at the output.

In general, the system response can be considered lineal, and thus only one or two constant factors per fiber are required to define the correction necessary for the fibers. From a mathematical point of view, these factors represent the slope and the intersection of the straight line at the source which best approximates each fiber's response. For the sake of simplicity, only one correction parameter and gray-tones image processing will be considered in the present article. The general structure proposed for the reconstruction table (RT) is shown in Table 1. This structure is that which will be necessary in order to be able to decode any image captured by the sensor.

The first two elements of the RT, r (*i*) and c (*i*) are obtained through a method for locating circular pattern images applied to a bundle image when it is homogeneously illuminated without reaching saturation. The results of this search are the first data to be included in the RT, together with the correction factor α(*i*). A fast and simple method that obtains good results has been described in [6]. The values R (*i*) and C (*i*) in Table 1 are obtained from subsequent processing of all the images captured by the sensor during the input scanning procedure. This operation implies verifying and analyzing the state of all the fibers in each image captured by the camera, respecting their order of appearance.

If each of the points contributed by the fibers relocated, according to the RT, we would obtain an image which we will call the *primitive image* (*Ip*). This image, although intelligible, will present a large number of empty regions that correspond to regions without fibers at the input (interstices), and to a lesser extent to the omission of some real fibers due to possible failures in location (see Figure 3).

Depending on the scanning method used, a specific decodification procedure should be applied. We know that each cell at the input end occupies a determined area. This is a discontinuous representation of the input exploration domain. In other words, one, and only one cell from the imaginary grid will be assigned to each fiber at the input end of the *IOFB*, according to its degree of proximity and influence. The number of cells to take into account in the scanning space depends on the maximum number of fibers (theoretical) that can be aligned in both dimensions, and their area is related to the nominal diameter of the fibers (d_fib_). The width w of the smallest line projected should satisfy the following expression:

(1) $${\text{d}}_{\text{fib}}\geq \text{w}\geq {\text{w}}_{\min}$$

where w_min_ refers to the minimum width of a line projected onto the input that is capable of exciting a fiber sufficiently (it has been empirically determined that this should cover at least more than 50% of its area). This working range guarantees that the response of any illuminated fiber can be distinguished from an unexcited state. Furthermore, a line with a width greater than that specified does not guarantee greater excitation because the *radiance* R_i_ that a particular fiber can transmit depends directly on the degree of superimposition of the line on the facet of the fiber more than on its width, such that:

(2) $${\text{R}}_{\text{i}}:\Leftrightarrow {\text{A}}_{{\text{fib}}_{\text{i}}}\cap {\text{W}}_{\text{f}}$$

where A_fibi_ represents the area of the fiber and W_f_ is the width of the line projected onto the fiber. The size of the final reconstructed image is defined by the range of the scanning space and this, in turn, depends on the maximum number of fibers (*nfib*_max_) that can appear aligned in any dimension. To determine the integer value, the following equation is used:

(3) $$\text{nfi}b_{\max}\approx \frac{B_{\text{diam}}}{F_{\text{diam}}}$$

where *B_diam_* is the diameter of the bundle and *F_diam_* is the nominal diameter of the individual fibers. This value is approximate given that the fibers are considered perfectly aligned.

### 2.2. Proposal for Scanning Using Differential Binary Space Encoding (DBSE)

**Figure 4.**
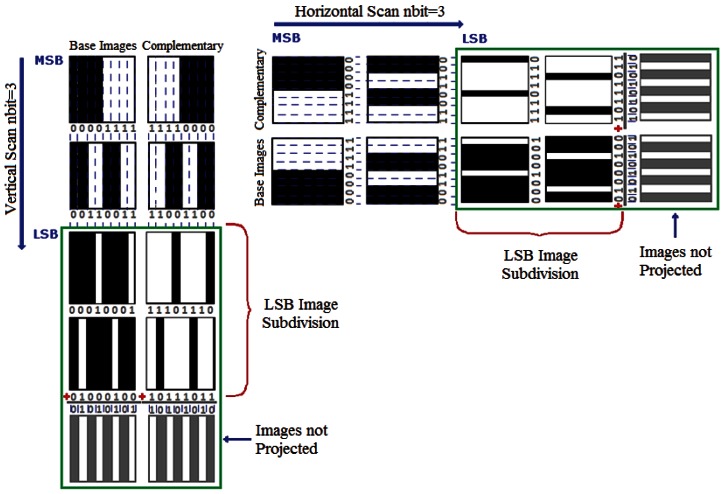
Pattern images for a DBSE scan with a number of bits *nbit* = 3. Note that the last image (LSB) of each dimension is subdivided into two.

Below, we describe the necessary procedures proposed for calculating the parameters included in the RT. The structure of each pattern image generated for scanning is conditioned by a base weighted binary code. However, in this study an alternative is proposed aimed at minimizing the problems discussed in Section 1 arising from the optical resolution of the system. The method, which we will call Differential Binary Space Encoding (DBSE), carries out differential processing of the images captured without implying an excessive increase in the number of images to process. Differential processing of the images implies that for each base pattern image, another, complementary pattern image is generated (see Figure 4). This ensures that a fiber illuminated by a base pattern image will be extinguished by its complementary image. If a fiber maintains its excitation slightly in the presence of both pattern images, it is considered extinguished since it has not undergone an appreciable change of state and the condition analyzed is not conclusive.

**Figure 5.**
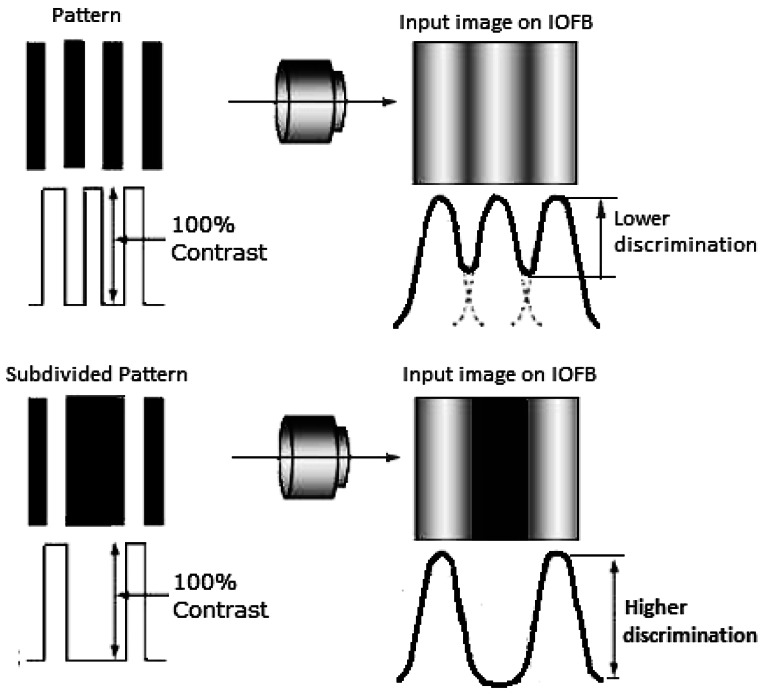
Subdividing the LSB pattern image constitutes an artificial means of using an optical system with lower optical resolution.

To understand this situation, the following example may help. Supposing that a region with light-dark transitions originating from the pattern image impacts on a fiber in such a way that nearly half the diameter of its nucleus is covered. In this case, the level of excitation registered in the fiber will be very similar both for the base image and for its complementary image. Therefore, the fiber is not considered to have changed its state. Another change that has been introduced concerns the structure of the last image of the sequence. This is associated with the least significant bit in the code and is formed by the thinnest lines (of alternate color) in the sequence, thus presenting the greatest frequency of change compared to the remainder of the images. Under these conditions, it is probable that the optical resolution of the system will be compromised and will not be appropriate for this type of scanning. In this case, the optical system can project a gray tone onto the IOFB rather than an image formed by lines, affecting the decoding process of the least significant bit in the code. To solve this problem, we opted to subdivide the pattern image corresponding to the least significant bit of each dimension into two images with their respective complementary images (see Figures 4 and 5).

As regards the method described in [8], where before for 256 × 256 fibers 16 images were required for the two dimensions, now 36 images will be required. Of these, nine are differential pairs for each dimension (18 + 18 = 36 images). Although the number of images rises, it remains lower than the number required for the line scan described in [6] for which, under the same conditions, the same scan space required a minimum of 512 (2 × 256) high resolution images for decodification.

### 2.3. Calculation of the RT

**Figure 6.**
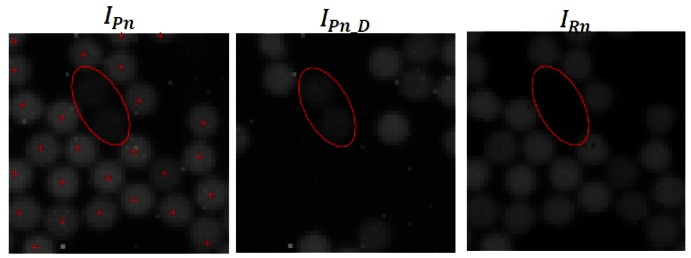
Example showing the discriminatory effect of the differential pattern images. The ellipse indicates two apparently illuminated fibers. Due to the ambiguity of their state they are considered “unlit”.

The RT construction process is similar to the calibration method using lines described in [6] and the structure of the tables remains the same. To complete the RT, the images captured by the camera are loaded into the memory maintaining their order of appearance, and a subtraction between each pair of differential images is carried out according to the expression:

(4) $$I_{\text{Rn}}=\left\{\begin{array}{l} I_{\text{Pn}}-I_{\text{PnD}},I_{\text{Pn}}>I_{\text{PnD}}\\ 0,I_{\text{Pn}}<I_{\text{PnD}} \end{array}\right\}$$

where I_Rn_ is the image resulting from the subtraction of the complementary images with subscript “*n*” captured by the camera. The subtraction operation enables us to reject the fibers that, in response to a differential pair of images present an “indeterminate” response because they are physically located in the middle of a light-dark transition border or *vice versa*. For example, if it is expected that a fiber exposed to a determined illuminated area of a base incident image will be illuminated, then it should be extinguished when presented with the complementary image and *vice versa*. In contrast, if the fiber is illuminated both by the base image and its complementary image, it can be stated that it presents an indeterminate behavior because the state of the fiber is not known with certainty. A case such as this is indicated with an ellipse in Figure 6.

It can be observed that fibers in an indeterminate state disappear, and only those fibers indicated by the symbol “+” in *I_Pn_* are considered excited. The advantage of using a set of differential images is that it helps to reject these cases where the fiber, despite having attained a certain degree of illumination, is not considered to have reached optimum excitation in response to a pair of specific pattern images.

For each resulting image, the state of the fibers is verified. In this way, a “position code” (a row or column, depending on the dimension analyzed) is “constructed” corresponding to each cell and this is stored in the RT. It should be noted that in order to determine the state of each fiber it is essential to know beforehand the central positions of the fibers, since it is from these positions that all the information used in the calibration analysis and for reconstructing the images is extracted. In our case, the procedure used for location was the FDDT (Fiber Detection using Distance Transform) technique described in [3,4], considering that all the fibers possessed a similar nominal diameter. To determine the state of a fiber in each of the resultant images, our proposal is to calculate the median gray level (or even the average) in the center of the fiber analyzed, using a set of nine pixels (N_9_ 3 × 3 pixels) such that:

(5) $$\{\begin{array}{c} I_{\text{MED}}\left(N_{9}\left(u_{c_{i}},v_{c_{i}}\right)\right)=\text{Med}\left(I\left(u,v\right)\right)\\ \forall (u,v):\{u_{c_{i}}-1\leq u\leq u_{c_{i}}+1,v_{c_{i}}-1\leq v\leq v_{c_{i}}+1\} \end{array}$$

If the average gray level exceeded a threshold value, the fiber was considered “illuminated” and was associated with the logical value “1” in the bit position code. The position of the corrected bit also corresponded to the order of appearance of the image analyzed. If it did not exceed the threshold mentioned, it was considered “unlit” and associated with the logical value “0” in the bit corresponding to the position code.

(6) $$\{\begin{array}{c} 0<I_{\text{MED}}>{\text{δ}}_{\text{GL}}\to “1”\to “\text{lighted}”\\ 0<I_{\text{MED}}<{\text{δ}}_{\text{GL}}\to “0”\to “\text{off}” \end{array}$$

To construct the numerical values Ri/Ci, the real state of each fiber was verified (illuminated-1/unlit-0) in all the images. The binary code corresponding to the row or column position was obtained from each state, respecting the order in which the images appeared. Having completed the analysis of all the fibers and all the images, the final result was a preliminary RT. The time taken to construct the RT is very low and few images are required for the analysis. Note that when 36 initial images (8 bits) are used, the number of images to store for subsequent analysis can be reduced by half due to the implicit subtraction operation.

### 2.4. Refining the RT in DBSE

**Figure 7.**
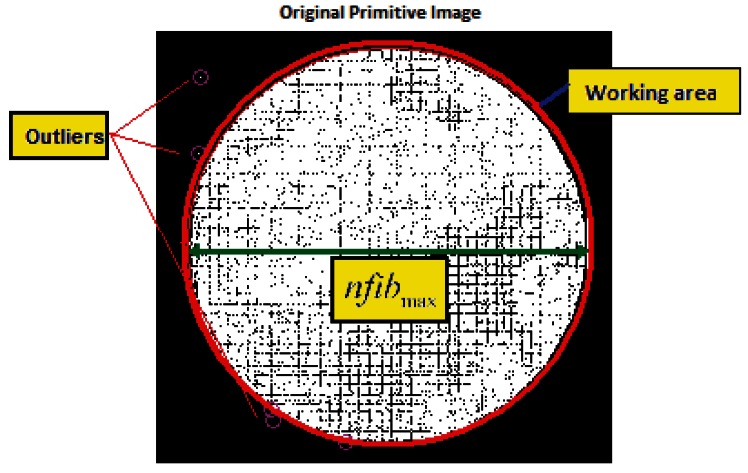
Confidence positions and atypical values (outliers) in a primitive image.

The RT should be refined to verify the possible occurrence of empty, duplicate entries, or entries with atypical values (outliers). This is fundamentally due to poor correspondence of the scanning area at the bundle input, or to errors in determining the state of the fibers that affect a bit during the construction of the position values Ri/Ci. *Outliers* are fundamentally due to poor focus or to false fiber detections, and their number is generally low or nonexistent if, prior to calibration, good focus and correspondence between the bundle and the calibration monitor has been ensured. Each cell position registered in the RT should comply with a physical model that is consistent with reality since no fiber is located outside of the physical limit imposed by the shape of the bundle. For this reason, each pixel in the primitive image should registered within a circumference, the center of which (*u*_0_, *v*_0_) is the center of mass for all the cell positions calculated (Figure 7). Consequently, all values considered atypical should be eliminated from the preliminary RT. The maximum distance (confidence circle) is directly related to the maximum number of fibers considered in the scan, such that:

(7) $$\text{nfi}b_{\max}\approx 2\sqrt{{\left(u-\text{uo}\right)}^{2}+{\left(v-\text{vo}\right)}^{2}}$$

To analyze the coordinates of redundant cells, it is first necessary to identify them in the RT and subsequently to determine which is “the best” or “the most appropriate” of the entries which present conflicts. A simple means to locate them is to order the RT entries by cell position. In this way, the redundant entries “disputing” over the same cell, are grouped consecutively and are thus easier to process. Each group of entries is analyzed separately. For each group in conflict, “the best” entry is chosen. In other words, the entry that is closer to an ideal condition will remain unaltered in the RT. The remainder should be relocated toward empty, neighboring cells that have not been included in the RT (if there are any). If any entry cannot be reassigned then it is eliminated from the RT. In order to correct the RT, all the gray levels registered in response to each pair of pattern images must be analyzed again for each fiber.

The “best entry” from a group disputing over the same cell is the one closest to an ideal condition. However, what is an ideal condition? We considered that ideal fiber excitation (or an ideal condition) existed when each time the fiber was illuminated from the input, it attained its maximum level of light transfer and, on the other hand, when it was unlit it reached the minimum degree of intensity at the output. If these ideal conditions always occurred in the fiber, this would indicate that each fringe exciting produced the maximum superimposition on its nucleus at the input, and, in contrast, when it was unlit it would indicate that it was not receiving any influence.

Normally, this does not always occur; a fiber is more or less illuminated depending on the degree of the fringe superimposition on its nucleus. However, bearing in mind the sequence of gray levels that should be obtained under ideal conditions and comparing it with the real sequence, an idea is obtained of the extent to which the result obtained resembles that sought. In other words, the ideal condition serves as a reference for comparing the different entries of a group in dispute and defining which is the best candidate for that cell.

When the maximum gray level $\left(g_{\text{max}}^{i}\right)$ reached by each fiber during the scan is known, a pattern values vector (or pattern chain) can be constructed from n bits by means of:

(8) $$\text{Cp}\to \left[b_{n-1}\cdot gi_{\max},\ldots \ldots .b_{2}\cdot gi_{\max},b_{1}\cdot gi_{\max},b_{0}\cdot gi_{\max}\right]$$

where *b_k_* is the weight {∀*k* = 0, 1, .., *n*} which has the value of 1 for the “illuminated” fiber and 0 for the “unlit” fiber, and $g_{\text{max}}^{i}$ is the maximum level of gray that has been registered for the fiber *i* by the sensor. This representation is analogous for rows and columns, and thus each fiber will have its own pair of ideal sequences. Similarly, considering $g_{k}^{i}$ as the average real level of gray reached by the fiber *i* in the image *p* = {0, 1, 2, n − 1}, then, for each redundant entry we obtain the vector:

(9) $$\text{Cr}\to \left[b_{n-1}\cdot gi_{n-1},\ldots \ldots .b_{2}\cdot gi_{2},b_{1}\cdot gi_{1},b_{0}\cdot gi_{0}\right]$$

To analyze the degree of similarity between the pattern and redundant chains, the quadratic error produced is calculated. The root of the mean quadratic error for each real code compared to the pattern is:

(10) $$\text{RMSE}=\sqrt{\frac{\overset{p=n-1}{\underset{p=0}{\text{∑}}}{\left(b_{p}{\text{gi}}_{\max}-b_{p}g_{p}\right)}^{2}}{n}}$$

The combination giving the least error out of the redundant cell combinations is chosen and remains in the TR. The remainders of the redundant entries are relocated toward the positions of neighboring cells not registered in the RT, where errors between pattern chains *Cp* (row and column) and *Cr* are also minimized. These values are temporarily stored and verified again to check whether new redundancies appear when all entries are verified again. If, after a specific number of iterations, not all cases have been solved, these are definitively eliminated from the RT, since they may be associated with false fiber detection, and where these arise, their number is very low compared to the remainder of validated entries.

### 2.5. Experimental Setup and General Considerations

The results reported in this article were obtained using a software application built in Matlab containing all the operations necessary to conduct a spatial calibration of the system and to evaluate both the line scan method and the DBSE. The application was run on a Pentium Core 2 Duo 3 GHz 4 GB RAM PC. A monochrome BCi4-6600 camera was used with a 6.6 megapixel CMOS sensor and a 2,208 × 3,000 pixel matrix. The optics used was a 19–35 mm optical zoom from Cosina. The sensor and the screen were isolated into a dark box to prevent external influences (for example: sunlight, artificial lighting variations and reflections on the screen, *etc.*) Camera resolution was established based on the assumption that each fiber occupied an effective area of around 7 × 7 pixels, in order to ensure adequate location of the fiber in the output image.

An AOC TFT screen (17″) was used with a resolution of 1,280 × 1,024 and pitch size of 0.064 mm. This device should be perpendicular to the bundle input end in order to avoid errors and distortions in the calibration caused by inadequate perspective. All experiments were conducted using a plastic fiber bundle 2.8 m in length and containing approximately 50,000 fibers with a nominal fiber diameter of 50 μm [11]. Given these characteristics, *nfib*_max_ in Equation (7) was approximately 256 fibers in both dimensions.

In accordance with the geometry of the installation, an active screen area of 768 × 768 pixels was chosen, which implies that w in Equation (1) was 3 pixels wide.

**Figure 8.**
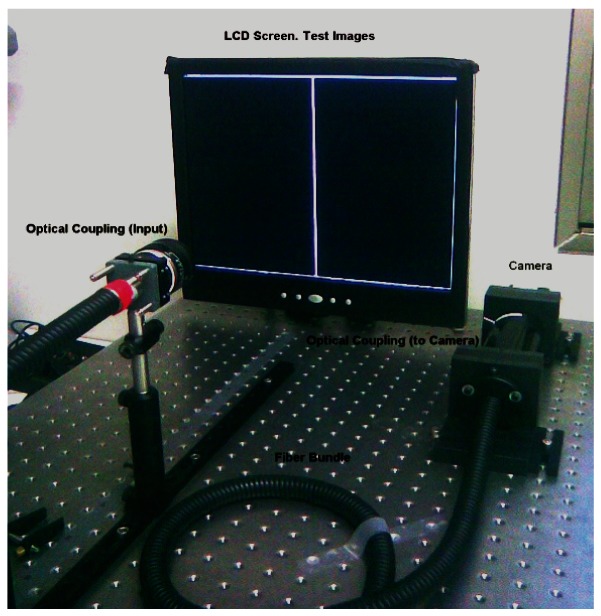
System setup.

Figure 8 depicts a general overview of the experimental setup used. This study has been based on [2,3,6], the algorithm for fast fiber location (FDDT), the focus method using *f_var_* measurement and the line scan method employed as a reference scan method.

### 2.6. General Calibration Procedure

In order to obtain the law of correspondence between the input-output of the fibers (decoding), it is necessary to carry out a set of tasks run in the following sequence:

1. Correctly focus the bundle by means of the *f_var_* metric described in [2] and adjust the position of the bundle input end, so that, it will completely capture the active area that the pattern images will occupy, in order to optimize the scanning space.
2. Locate all the fiber positions in an image captured by the camera. This is carried out by means of a FDDT algorithm and an image of the homogeneously illuminated bundle, which enable rapid location of the fiber centroids.
3. Determine the equalization factors which will compensate for the fiber responses.
4. Once the entire system has been adjusted, the encoded images should be exposed sequentially and, at the same time, each image captured by the camera should be captured and stored in well-differentiated files.
5. For each resultant image, the fibers previously located using FDDT and showing a great lighting excitation will be stored in a table. This operation makes it possible to generate a binary position code for each fiber by dimension. These results are stored in a preliminary RT in the pair (R (*i*), C (*i*)).
6. Once the preliminary RT has been built, RT refinement is carried out to eliminate the outliers and the redundant coordinates. Once the system has been calibrated and the RT refined, it is necessary to verify that calibration is correct.

**Figure 9.**
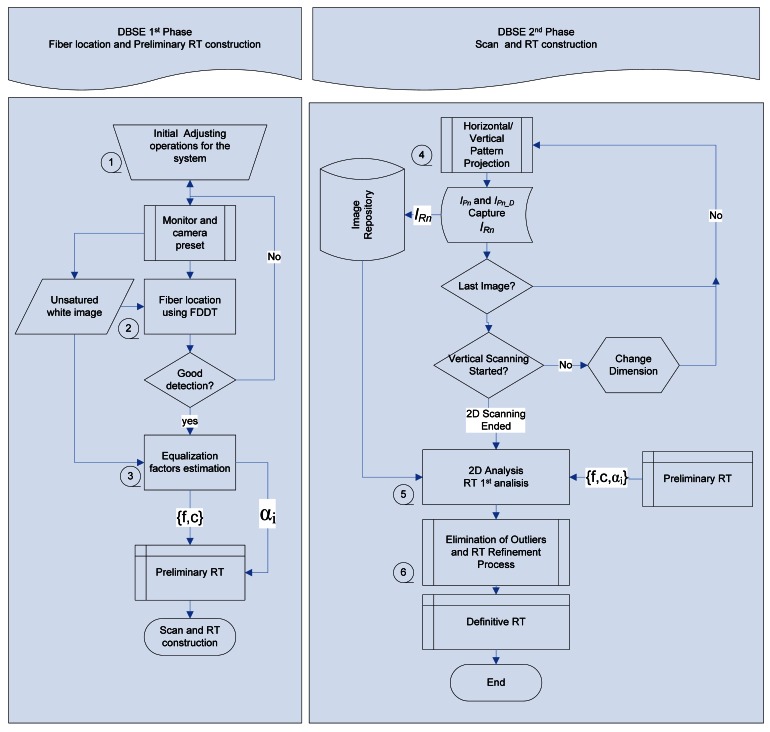
Flow chart of the procedure to follow in DBSE.

Figure 9 summarizes the steps listed above, subdividing the entire process into two phases. Each corresponding step number is also indicated. The first phase focuses on preparation of the system for calibration (focus, camera adjustment, *etc.*) and determination of fiber position and equalization factors based on a white image captured by the sensor without causing saturation. From this phase a provisional RT is obtained in which associations with cell positions are still to be determined.

The second phase consists of scanning with differential images and capturing the resultant images. Subsequently, all the images are analyzed to complete the RT, outliers are eliminated, and the results are refined. Once this phase is completed, the definitive RT is ready.

## 3. Results and Discussion

Regarding the previous studies used as a reference, it is difficult to compare some of the results obtained since not all the information about the original experiments which would be necessary is available. For this reason, the results reported here were obtained respecting the general ideas described but adapting them to the specific conditions of the experimental setup.

**Figure 10.**
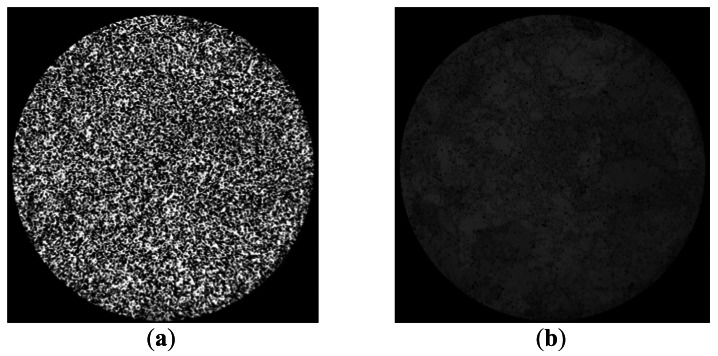
Influence of input focus on the same image formed by alternate black and white lines captured by the sensor. (**a**) focused and (**b**) not focused.

Before carrying out calibration using the techniques that will be analyzed in this section, we applied the focus methodology of the optical system proposed in [2], employing a metric based on variance in the gray levels contributed by the fibers. This step was essential to obtain correct spatial calibration since calibration methods based on space encoding are especially sensitive to this aspect because, in contrast to the line scan technique, the light-dark frequency change rises progressively with scanning. Figure 10 shows the effect that a poorly focused input optic would have on the image obtained by the sensor (disordered). The input pattern image used corresponds to the least significant bit formed by alternate black and white lines which are three pixels wide per line on the screen (worst case scenario).

In Figure 10(a), the image is well focused whereas in Figure 10 (b) it is not, and therefore the image captured tends to be more homogeneous due to energy scattering at the input, indicating that significant errors may be produced during RT calculations. In this case, decodification of the least significant bit would be affected. If an error were produced in the least significant bit, the error in calculation of position would be much greater.

Table 2 shows a comparison of line scan calibration methods, the space encoding techniques described by Dujon [8] and the differential method proposed here. These results were obtained under the same working conditions in terms of camera configuration, hardware, lighting, *etc*.

As can be seen, the method which requires most time for scanning and processing is the luminous line scan technique, which requires a much higher number of high resolution images (in our case, 6.6 megapixels) to be processed if the space encoding techniques of Dujon and DBSE were used instead. This implies massive memory use for image storage, as well as notable use of the system's operating memory, both aspects which require adequate management. Nevertheless, the results obtained are very good, with a high number of entries being validated after the RT refinement procedure.

It can also be observed that the number of entries deleted in the analysis, or entries to which it has not been possible to assign a coherent position at the input (*outliers)*, is smaller. This question is related to the fact that position error does not depend on the reconstruction of a binary position code, as is the case with space encoding, but rather, it depends on the level of certainty about the position of maximum excitation for each fiber. This error is generally in the range of ±1 positions for each dimension.

Methods based on space encoding are quantitatively superior to the line technique regarding processing speed, memory use for storage and post-processing of the images captured, fundamentally as a result of the reduction in the number of images involved. Both the Dujon and DBSE methods described above achieve a high number of validated entries compared with the number of initial entries included in the RT, although, not always as many as the line scan method. Nevertheless, it can be seen that in both cases the quantity of entries assigned is very high (>80%), and it is possible to reconstruct good quality images in accordance with the maximum number of bundle fibers.

It is to be expected that in order to obtain good results with space encoding techniques, a higher resolution optic is required. If the system does not possess the necessary focus and optical resolution, the number of outliers may increase notably because the number of errors in the position codes estimated would also rise. It is precisely regarding this aspect where DBSE has proved to be superior to the technique described by Dujon, and thus can serve as the basis for future research. Differential image processing provides greater immunity to calibration errors, showing a significant increase in the number of validated entries compared to the method described by Dujon. This improvement is mainly due to the usage of complemented patterns images, FDDT and the redundancies analysis, which allow discarding undetermined states of the fibers during the RT calculation.

Furthermore, in order to reduce the appearance of errors due to this fact without using a very expensive input optical system, the results can be further improved by creating a subdivision of the pattern images associated with the least significant bit in the position codes. In this way, a reduction in the frequency of change in the pattern images is artificially obtained. However, this inherently implies an increase in the number of images to process, although this will always be much lower compared to the line scan calibration technique.

On the other hand, the method described by Dujon presents an additional difficulty related to the procedure for determining the state of excitation of the fibers in response to each pattern image. An excitation threshold is used which is determined by an iterative optimization procedure that can increase calibration time. In contrast, DBSE discriminates the indeterminate states of fibers, and thus the optimum threshold that serves as a reference for determining the real state of the fiber is zero (or very close), implying notable savings in terms of time.

**Figure 11.**
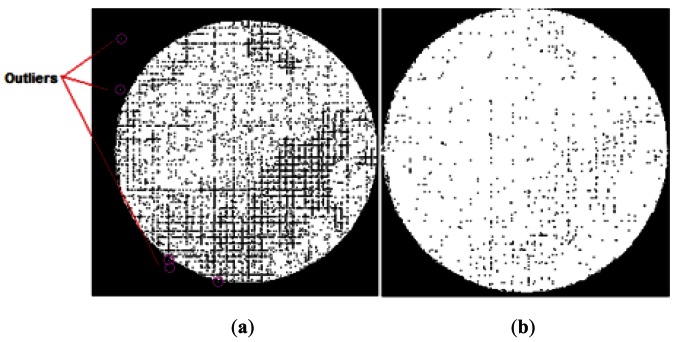
(**a**) Original primitive image, (**b**) Primitive after RT redundancy correction.

The analysis of redundancies makes it possible to relocate a specific number of positions that share the same cell in the RT (redundant registrations) toward empty pixels. This is another of the main characteristics that distinguish DBSE, from Dujon method. Figure 11 shows the evolution of a primitive image (and therefore, of the RT) corresponding to a totally white input image, when the redundancy correction analysis is applied using the DBSE method. Figures 11(a) and 11(b) represent, respectively, the initial state of the primitive image, after a first scan analysis, and following the redistribution of redundant positions in the RT and elimination of the *outliers*. This procedure ensures that each represented pixel is in a “probably optimum” position, and covers a greater area of the circular shape of the image, facilitating a subsequent *inpainting* procedure.

*Inpainting* is an essential procedure to achieve correct reconstruction of the final image that is consistent with the original input structure, and techniques based on calculation of variance, PDEs and mask convolutions, *etc.*, are usually employed for this purpose. However, this procedure will not be analyzed in this article as it falls outside the main area of interest, although it is interesting to give some examples of reconstructed images obtained in uncontrolled environments.

**Figure 12.**
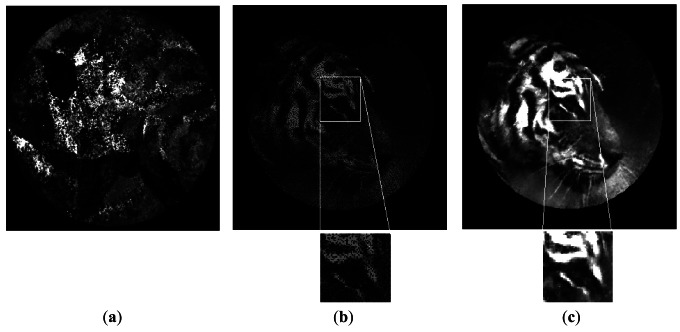
Image Progression and details. (**a**) Sensor Image, (**b**) Primitive Image, (**c**) *Inpainted* Image.

Figure 12 shows an example of the evolution of an image transmitted through a sequence of images. Initially, the image captured by the camera is shown in (a), subsequently the corrected primitive image is presented in (b), and finally in (c), the completely reconstructed image using the inpainting technique described by Oliveira is given [12]. The primitive image is formed by extracting the gray levels contributed by the located fibers, and subsequently reordering and equalizing the information (a) according to the RT.

**Figure 13.**
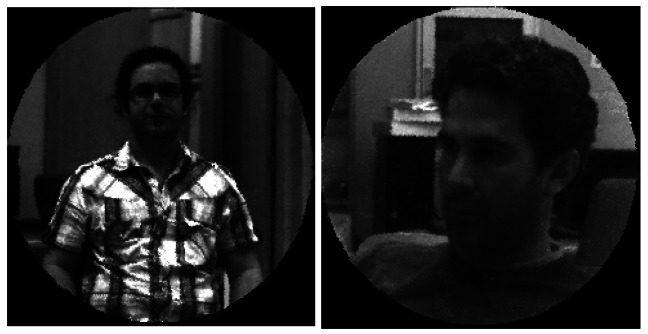
Real images captured with the experimental system.

Figure 13 shows another two real examples captured by the system. The results were obtained after carrying out a DBSE calibration; it can be seen that the images present good contrast and the quality is appropriate for the spatial resolution of the system presented here, where the images did not exceed 254 × 254 pixels.

**Figure 14.**
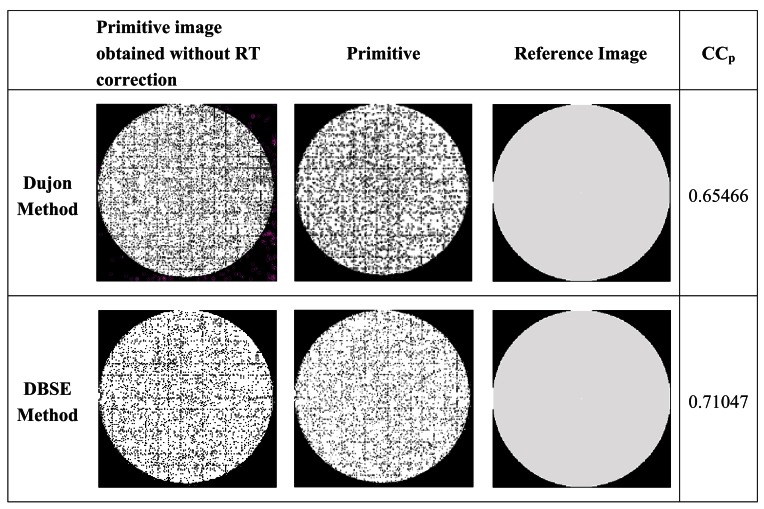
Primitive images and reconstructed images decoded using the RT for BSE and DBSE, respectively.

In order to demonstrate the improvement achieved by using DBSE compared to the technique described by Dujon. Figure 14 shows a sequence of images for each technique, corresponding to the initial primitive images (blank), the corrected images and lastly, a reference image in white. Note the significant decrease in the number of interstitial spaces and outliers (in magenta) in the primitive images corresponding to the DBSE, with respect to the Dujon method. So, this indicates the improvement obtained by DBSE respect to Dujon. Calculating the correlation coefficient (CC_p_) between the primitives and the reference image, the improvement obtained by DBSE is demonstrated.

In [7], system calibration was carried out by means of two techniques, the single-mode fiber with pixel block scanning and that of Dujon. However, this study did not employ fiber location or any appropriate focus method. The information that was extracted from the fibers was not calibrated for intensity and the RT was constructed on the basis of the illumination changes present in each image resulting from scanning; thus it is to be expected that the number of calibration errors would be very high, generating a large quantity of outliers.

**Figure 15.**
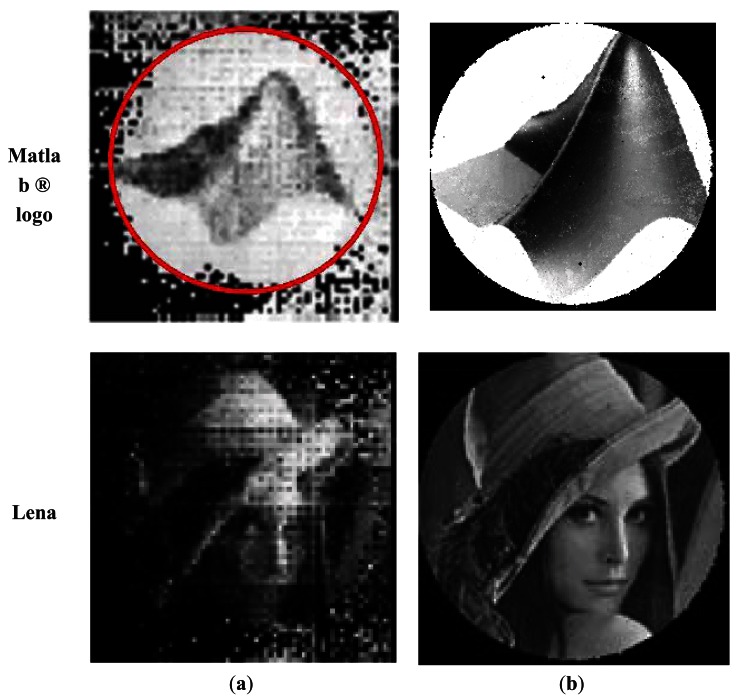
Comparison of results obtained using the method described in [7] and a modified version using DBSE. (**a**) Dujon method implemented and discussed in [7], (**b**) Reconstruction using DBSE.

Figure 15 reproduces some of the results obtained in [7] with respect to those obtained in this study using the logo of Matlab ® and the Lena image. The advantage of using fiber location and differential pattern images is clearly evident. Unfortunately, it is not possible to present comparative results that better illustrate these differences.

**Figure 16.**
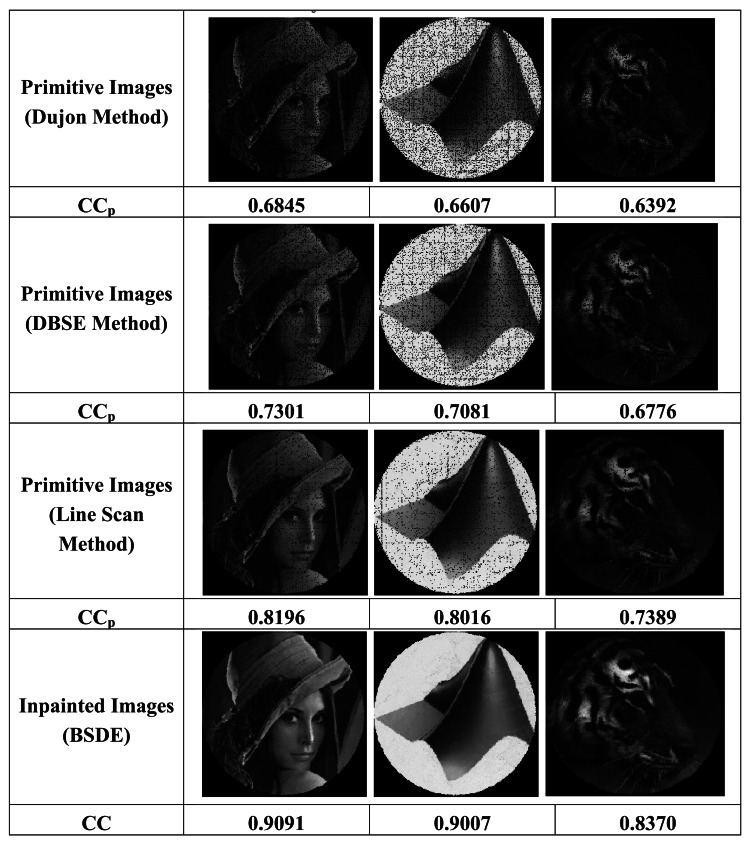
Images reconstructed using different calibration methods.

Figure 16 shows some primitive images obtained by means of the techniques analyzed in Table 2. In order to quantify the quality of the results, two correlation coefficients were calculated. The former (CC_p_), estimates the similitude between each primitive and its original image. The second one (CC), calculates the correlation between an *inpainted* image and the original one (not shown). CC has been calculated only for DBSE method, because it would be practically the same for the rest of the methods due to the reduction of the number of interstitials interpolated by the *inpainting* procedure. This value is really near to 1 indicating a good degree of similitude between the *inpainted* image and the original one. Note the significant decrease of the number of interstitial spaces in the primitive images corresponding to the methods of DBSE and Lines, with respect to the method of Dujon. The coefficients CC_p_ clearly demonstrate the improvement reached in the primitive images for DBSE and Line methods, with respect to Dujon.

## 4. Conclusions

In this article, a new technique has been presented for calibrating image transmission systems based on IOFBs. We have demonstrated that transmission via IOFBs is an alternative to other, technologically consolidated fiber-based elements such as coherent bundles. The DBSE calibration technique presented here is based on the space encoding technique, but the principal contribution is on the use of differential pattern images. For the purposes of comparison, we have taken the luminous line scan technique and the method developed by Dujon [8] as references to compare and validate the proposed calibration model, using a very simple experimental setup.

The experiments showed that all the proposed methodology is valid and that it is capable of offering good results; however, it is necessary to highlight the need to ensure certain aspects:

- Adequate fiber location, an aspect which has been effectively solved using the FDDT technique described in [3,4].
- Correct focus of the optic at the input, which can be achieved by means of the *f*_var_ metric described in [2].
- Resolution of the input optic can condition the application of space encoding techniques.

From the experiments that have been presented, the following can be concluded:

- DBSE is a valid proposal since it achieves good image quality and is faster than the line scan method. However, the line scan method achieves better results since it generates less ambiguous results or *outliers*. However, DBSE is a strong method whenever sufficient optical resolution of the system can be guaranteed.
- The results given in Table 2 show that validation of the RT entries is over 80% compared to the initial entries obtained with FDDT. This guarantees good quality in reconstruction of the final and primitive images since less than 1.59% of the information is lost through calibration errors.
- The redundant coordinate correction procedure enables redistribution of most of the ambiguous cases towards other, more optimum positions, providing a notable improvement in the active pixel area of the image formed.
- We have shown that image focusing strongly influences calibration, and the DBSE method is the most sensitive to this effect. This problem can be minimized by using other, alternative base codes to generate the pattern images, and this will be the subject of future research.
- Of the techniques taken as points of reference, the technique described by Dujon presents the worst results in terms of quality of the reconstruction, discrimination of the state of the fibers and in the generation of outliers.
- The luminous line scanning method continues to represent a more accurate alternative to DBSE. Nevertheless, the results are not very different and in no instance was the quality of the final image seen to be compromised. With the DBSE method, the reduced use of storage and processing memory is notable, as is the greater speed.

In future research, the results will be extrapolated to a system with a lower resolution sensor, in order to be able to conduct high resolution calibration for applications using less expensive sensors which offer the same functionalities.

## Figures and Tables

**Table 1.** General structure of the RT.

r(i)		c(i)	α_i_	R(i)	C(i)
where:					
(r(*i*), c(*i*))	→	Coordinate pairs of the fibers located by the sensor.			
α*_i_*	→	Intensity equalization factors.			
(R(*i*), C(*i*))	→	Position of the cell that best excites a fiber in (*r*(*i*), *c*(*i*)).			

**Table 2.** Comparison of the different calibration techniques analyzed.

**Parameters**	**Method**		

	**Line**	**Dujon**	**DBSE (8bits)**

Number of fibers located. Initial RT entries	49,127	49,127	49,127
Final validated entries	46,454 (94.5%)	40,241 (81.9%)	42,711 (86.9%)
Corrected entries (redundant)	3,270	1,920	5,867
Eliminated entries	2,672 (5.4%)	6,077 (14.1%)	6,416 (13%)
Mean scan time	7.91 min	2.6 min	5.54 min
Mean RT calculation time	38.94 min	1.3 min	2.2 min
Mean analysis time of redundancies and outliers	13.98 min	2.5 min	5.36 min
Number of images used	522 *	16	36
Final image size [pixels]	261 × 261^1^	254 × 254	254 × 254

* A scan space of 261 × 261 images in each dimension was considered. This inflated size of the grid is subsequently corrected in the TR so that the size of the image is not greater than *nfib*_max_ = 256 in each dimension, eliminating those cell positions that do not have an appreciable influence on the fibers.
